# Hydrogen Sulfide Recruits Macrophage Migration by Integrin β1-Src-FAK/Pyk2-Rac Pathway in Myocardial Infarction

**DOI:** 10.1038/srep22363

**Published:** 2016-03-02

**Authors:** Lei Miao, Xiaoming Xin, Hong Xin, Xiaoyan Shen, Yi-Zhun Zhu

**Affiliations:** 1 Department of Pharmacology, School of Pharmacy and Institutes of Biomedical Sciences, Fudan University, Shanghai, China; 2 Department of Pharmacology, School of Pharmacy, Macau University of Science & Technology, Macau, China

## Abstract

Myocardial infarction (MI) triggers an inflammatory reaction, in which macrophages are of key importance for tissue repairing. Infiltration and/or migration of macrophages into the infarct area early after MI is critical for infarct healing, vascularization, and cardiac function. Hydrogen sulfide (H_2_S) has been demonstrated to possess cardioprotective effects post MI and during the progress of cardiac remodeling. However, the specific molecular and cellular mechanisms involved in macrophage recruitment by H_2_S remain to be identified. In this study, the NaHS (exogenous sources of H_2_S) treatment exerted an increased infiltration of macrophages into the infarcted myocardium at early stage of MI cardiac tissues in both wild type (WT) and cystathionine-γ-lyase-knockout (CSE-KO) mice. And NaHS accelerated the migration of macrophage cells *in vitro*. While, the inhibitors not only significantly diminished the migratory ability in response to NaHS, but also blocked the activation of phospho-Src, -Pyk2, -FAK^397^, and -FAK^925^. Furthermore, NaHS induced the internalization of integrin β1 on macrophage surface, but, integrin β1 silencing inhibited macrophage migration and Src signaling activation. These results indicate that H_2_S may have the potential as an anti-infarct of MI by governing macrophage migration, which was achieved by accelerating internalization of integrin β1 and activating downstream Src-FAK/Pyk2-Rac pathway.

Myocardial infarction (MI) occurs, resulting in an inadequate substrate or oxygen supply of the downstream myocardium, and further leading to cardiomyocytes deterioration^1^. These processes are mediated by a wide array of inflammatory reactions or factors^2^. Monocytes/macrophages, the major source of the inflammatory factors, are of central importance for healing after MI^3^,^4^. Macrophages reside in both healthy and injured heart, and increase in number during disease^5^,^6^. And macrophage is a primary responder cell type that involved in the regulation of post-MI wound healing at multiple levels^2^,^4^.

Induced by MI, macrophages migrate into the infarct zone, initiate intracellular signaling, which localizes the inflammatory response to clean the debris and subsequently induce scar formation^7^. The infiltration of macrophages into the infarct area early after MI, is critical for infarct healing, vascularization, and cardiac function^8^. Early depletion of infiltrating macrophages impaire wound healing, provoke adverse left ventricular (LV) remodeling, and increase mortality after MI^9^. Injection of human activated macrophage suspension early after rat MI, promotes recruitment of resident macrophages and accelerates vascularization, tissue repair, and improves cardiac remodeling and function^10^. Therefore, manipulating macrophage migration and function could be a promising therapeutic strategy in optimizing the process of infarct repair.

Hydrogen sulfide (H_2_S), as a gasotransmitter, has been demonstrated to possess cardioprotective function in various models of cardiac injury^11^,^12^. CSE (cystathionine-γ-lyase) is the predominant H_2_S-generating enzyme in the cardiovascular system, and its deficiency significantly attenuates endogenous H_2_S and results in exacerbating myocardial ischemia/reperfusion injury^13^,^14^. Our previous studies, together with others, have demonstrated that H_2_S plays diverse roles in protecting against cardiovascular diseases such as atherosclerosis, myocardial ischemia and heart failure^15^,^16^,^17^,^18^,^19^. Some mechanisms are considered to contribute to the cardioprotection of H_2_S, such as protecting cells against oxidative stress by increasing glutathione, promoting the translocation of the nuclear transcription factor Nrf2 to induce the activation of numerous detoxifying genes^20^. However, the influence of H_2_S in macrophage migration and the contribution to infarct repair have not been clarified. Thus, the objective of this study was to elucidate the impact of H_2_S on macrophage infiltration and/or migration after MI and investigate the involved mechanisms, to offer a new dimension to our understanding of macrophage recruitment post MI.

## Methods

### Reagents and antibodies

NaHS was administered instead of H_2_S. NaHS and the Pyk2 inhibitor (PF431396) were obtained from Sigma-Aldrich; The FAK specific inhibitor (PF573228), the Src inhibitor (PP2), and the Rac1 inhibitor (NSC23766) were purchased from Selleck Chemicals. CES-siRNA (sc-142618) was obtained from Santa Cruz. Rhodamine was obtain from Cytoskeleton, and DAPI from Beyotime; Following antibodies were used: anti-Rac, anti-Fak, anti-p-FAK^397^, anti-p-FAK^925^, anti-Src, anti-p-Src, anti-β-actin, anti-Integrin β1, anti-Integrin β3, anti-Cavenolin-1,anti-p-Pyk2 (Cell Signaling); anti-CSE (Santa Cruz); Anti-Integrin β1-FITC, anti-Galectin-3 (eBbioscience); anti-CD68 (Biolegend); Lactate dehydrogenase (LDH) assay from BeyotimeInstitute of Biotechnology.

### Animals

Mice devoid of CSE (KO mice) were normal in growth rate and reproduction, but had markedly reduced endogenous H_2_S production. WT and KO mice used in this study were littermates obtained via heterozygous breeding. The animal procedures were performed in accordance with the Animal Management Rules of the local authorities and were approved by the ethics committee of Experimental Research, Shanghai Medical College, Fudan University.

### MI Models

The left coronary artery was ligated permanently to induce myocardial infarction model as previously reported^17^.

### Immunohistochemical analysis

The hearts excised from sacrificed animals were fixed in 4% paraformaldehyde, sectioned at 5 μm following embedded in paraffin. The immunohistochemical staining was performed with an EnVision Kit (Dako, Carpinteria, CA). Antibody specific for macrophages (anti-CD68, Biolegend and Galectin-3, eBioscience) was used to selectively detect macrophages.

### Cell line culture

The murine macrophage cell line, RAW264.7, was purchased from American Type Culture Collection and maintained in RPMI 1640 medium supplemented with 10% FBS (HyClone, Logan, UT), at 37 °C in 5% CO_2_. The cells were treated with various concentrations of NaHS diluted in the RPMI 1640 medium (50, 100 and 200 mM) for different time points. Pharmacological inhibitors (5 μM of PF431396 or PF573228, 10 μM of PP2 and 25 μM of NSC23766) were treated for 1 hour before NaHS was added.

### Isolation of macrophages from bone marrow

Bone marrow-derived macrophages (BMMs) were isolated using standard protocols^21^,^22^. After differentiation for 7 days in RPMI-1640 containing 10 ng/ml recombinant murine M-CSF, cells were either untreated or incubated with NaSH for Co-culturing.

### Neonatal Mouse Primary Cardiomyocyte isolation

Primary cardiomyocytes were obtained from the ventricles of One- to two-day-old neonatal mice according to the method described^23^. The isolated primary cardiomyocytes were seeded into a 24-well plateat the density of 1*10^6^/ml then subjected to hypoxia for 4 h, in accordance with the technique described^24^.

### Co-culturing of BMMs and Cardiomyocyte

For transwell co-culturing, the 0.4-mm-pore size transwell insertscontaining 2*10^5^ BMMs were placed into the 24-well plate with cardiomyocytes that were hypoxia initially. Co-culture system was incubated for 12 h.

### Lactate dehydrogenaseassays

Lactate dehydrogenase (LDH) was detected to evaluate the severity of cardiomyocyte injury. LDH released in the culture medium was measured using commercial kits, according to the manufacturer’s instruction.

### Real-time PCR

For CSE gene expression analysis, total RNA was extracted from RAW264.7 cells using TRIzol (Takara) method. cDNAs were synthesized with the RevertAidtm First Strand cDNA Synthesis Kit #1622 (Fermentas). The primer sequences for the CSE were previously described^13^.

### Immunofluorescence and confocal microscopy

RAW264.7 cells plated onto glass coverslips were treated with various concentrations of NaHS for indicated time. The cells were fixed by 4% paraformaldehyde and blocked with 5% BSA (Amresco), following permeabilization by 0.5% Triton X-100. The cells were incubated with Rhodamine-conjugated phalloidin (red) for F-actin staining and DAPI (blue) for nucleus staining, or with primary antibody against integrin β1 followed by the incubation of appropriately labeled secondary antibodies. Confocal laser scanning was carried out with Zeiss710 confocal imaging system.

### Migration

The migration of RAW264.7 cells exposed to NaHS was determined by Transwell assays using polycarbonate transwell filters (Corning, 8 μm). Cells incubated in the presence or absence of NaHS, or pharmacological inhibitors were seeded into the upper compartment (containing 1% FBS, while the lower compartment contains 10% FBS only). The cells were allowed to migrate for 6 h before they were fixed in cold methanol. The non-migratory cells in the upper compartment were removed with a cotton swab, and the migrated cells were stained with 0.4% crystal violet (Sigma). For each experiment, the number of transmigrated cells in five random fields on the underside of the filter was counted and photographed, and three independent filters were analyzed.

### RNA interference

The siRNA to CSE (sc-142618) was obtained from Santa Cruz Biotechnology. And the siRNA to mouse integrin β1 were chemically synthesised by Shanghai GenePharma Co., Ltd. The siRNA sequences for integrin β1 were designed as follows: 5′-CAG AGA CAUUACUCAGAUdTdT-3′ (forward) and 5′-AUC UGA GUA AUG UCU UCU G dTdT-3′ (re-verse). A scrambled small RNA sequence was used as a negative control. The siRNAs were transfect into RAW264.7 cells in OPTI-MEM I Reduced SerumMedium (Gibco) for 24 or 48 h. The medium could be changed 6–8 h after transfection.

### Protein isolation

Total proteins or cell surface proteins of cells were extracted by M-PER Mammalian Protein Extraction Reagent (Pierce) and Pierce Cell Surface Protein Isolation Kit (Thermo Scientific) respectively according to the manufacturer’s instructions. Membrane proteins were extracted by Membrane and Cytosol Protein Extraction Kit (Beyotime). The protein concentration was quantified using a BCA protein assay kit (Beyotime).

### Western blot

The extracted proteins were separated and transferred to nitrocellulose membranes. The membranes were blocked, following immunoblotted with appropriate antibodies. Then, the blots were developed by Immobilon^TM^ Western Chemiluminescent HRP Substrate (Millipore). And the immunoreactivity was visualized with a chemoluminescence reagent.

### Statistical analyses

Quantitative data were presented as mean ± SEM. The calculations were performed with GraphPad Prism 6.0 software. Differences between two groups were analyzed by two-tailed Student’s t-tests, or assessed by one way analysis of variance with Tukey’s post-hoc test when more than two groups were compared. The differences were considered significant with P < 0.05.

## Results

### NaHS increased the infiltration of macrophages early after MI

**Figure 1:**
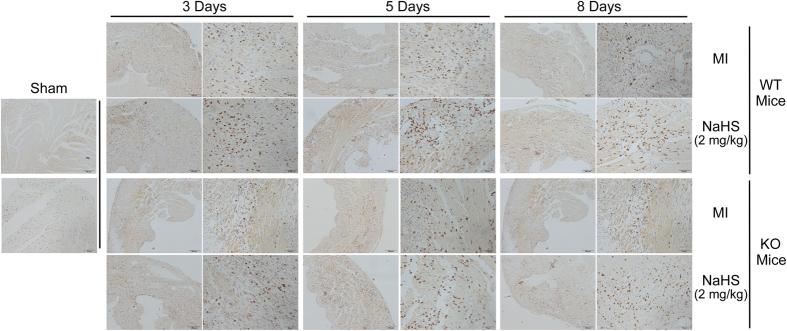
Myocardial immunostaining of CD68 after 3, 5, or 8 days of post-MI treatment with NaHS in both WT and KO mice. Scale bars, 200 μm and 500 μm.

Macrophage infiltrate into the myocardium is required for proper healing of infracted myocardium^8^. To identify the role of NaHS in cardiac remodeling, the recruitment of macrophages into the heart following MI was investigated by immunohistochemical analysis of CD68 and galectin-3, the macrophage markers. As shown in Fig. 1, the density of CD68 immunostaining in KO (CSE knock out)-MI mice was apparently less than that in WT-MI mice. In both WT and KO mice, NaHS treatment significantly increased the density of CD68 staining. Interestingly, the expression of the macrophage marker was increased by NaHS treatment in WT infarct LV at day 3, peaked at day 5, and weakened at day 8 post-MI. Moreover, in the KO mice, the NaHS treatment rescued the decrease of infiltrating macrophages caused by CES depletion, and exhibited a similar time course for CD68 staining. Similarly, the immunostaining tendency of galectin-3 was consistent with CD68 (Supplemental Fig. 1). These findings indicate that H_2_S promotes macrophage infiltration in the early, but not later stage of MI.

**Figure 2:**
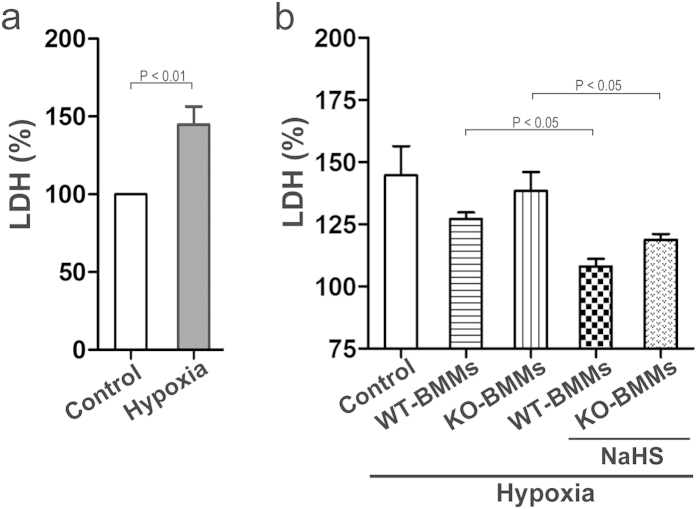
NaHS antagonized hypoxia-induced cardiomyocytesdamage. (**a**) The impair model of neonatal mouse primary cardiomyocytewas was developed, and the LDH in the cardiomyocyte supernatant was analyzed. (**b**) The BMMs were co-cultured with the cardiomyocytes in the NaHS presence or not, then the LDH in the cardiomyocyte supernatant was analyzed.

To test the potent cardioprotective efficacy of NaHS, the Lactate dehydrogenase (LDH) level in BMMs cell supernatant was determined in NaHS treatment assay. First, the isolated neonatal mouse primary cardiomyocytes were impaired by hypoxia, and LDH in supernatant was significantly increased, indicating a severity of cardiomyocyte injury (Fig. 2a). Then, BMMs, isolated from bone marrow of WT or KO mice, were co-cultured with the cardiomyocytes in the NaHS presence or not. As shown in Fig. 2b, although the LDH level was not apparently diminished in WT-BMMs and KO-BMMs groups, NaHS treated BMMs abrogated the LDH level largely compared to control group, demonstrating that macrophage infiltration triggered by NaHS antagonizes the damage of cardiomyocytes induced by hypoxia.

### NaHS enhanced the migratory ability of macrophages

**Figure 3:**
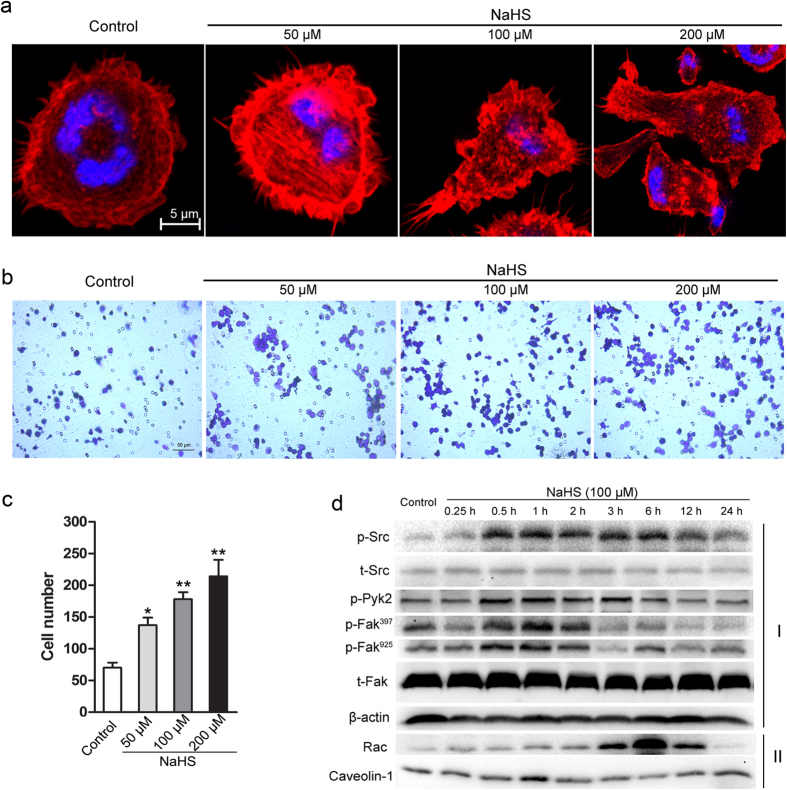
NaHS enhanced the migration capability of RAW264.7 cells. (**a**) RAW264.7 cells treated with indicated concentrations of NaHS for 6 h, then subjected to immunofluorescence analysis using Rhodamine-conjugated phalloidin (red) for F-actin, DAPI (blue) for nucleus. Scale bar, 5 μm. (**b**) After treatment with indicated concentrations of NaHS for 6 h, the migratory ability of RAW264.7 cells was analyzed by transwell assay. Scale bar, 50 μm. (**c**) The number of migrated cells per field was statistically analyzed. Values are the mean ± SEM from three independent experiments. *P < 0.05, **P < 0.01 versus control. (**d**) Western blot images of proteins from NaHS treated RAW264.7 cells, the probed antibodies as indicated (I, total cell lysates; II, membrane extracts; p, phosphorylated; t, total).

Cellular infiltration is suggested to be the migration of cells from their original sources. Given that NaHS preserved the cardiac function following MI through the increased infiltration of macrophages, it is still unclear whether and how NaHS stimulates macrophages to move from the border zone into the core of an infarct. To address these issues, the effect of NaHS on actin cytoskeleton was analyzed by immunofluorescent microscopy in the murine macrophage cells, RAW264.7. As shown in Fig. 3a, NaHS treatment resulted in significant rearrangement of the cellular actin cytoskeleton with the elongated lamellipodia, indicating a higher migration capability. The transwell assay also confirmed that NaHS dose-dependently increased the number of migratory RAW264.7 cells (Fig. 3b,c).

It is well known that Src/FAK axis played a pivotal role in macrophage mobilization, and Pyk2 and Rac activation were involved in the destabilization of endothelial cell contacts^25^. The Src-mediated FAK phosphorylation on several tyrosine residues plays an essential role in macrophage locomotion^26^. Thus, to ascertain the involvement of this signaling pathway in H_2_S-promoted macrophage migration, the phosphorylated status of Src, Pyk2 and FAK and activation of Rac in RAW264.7 cells were examined. As expected, significant increases in phospho-Src, -Pyk2, -FAK^397^, and -FAK^925^ were observed in RAW264.7 cells exposed to NaHS for 0.5, 1 or 2 h. However, total Src and FAK were unaltered. Additionally, membrane bound Rac, the active form of Rac in the down-stream of Src/FAK signaling, was also increased under NaHS treatment for 3 h, while the loading control Caveolin-1 in cell membrane was not altered (Fig. 3d). Thus, those results indicate that H_2_S indeed accelerates the migration of macrophages, and the Src-FAK/Pyk2-Rac signaling is involved in the process.

### NaHS triggered macrophage migration through Src-FAK/Pyk2-Rac axis

**Figure 4:**
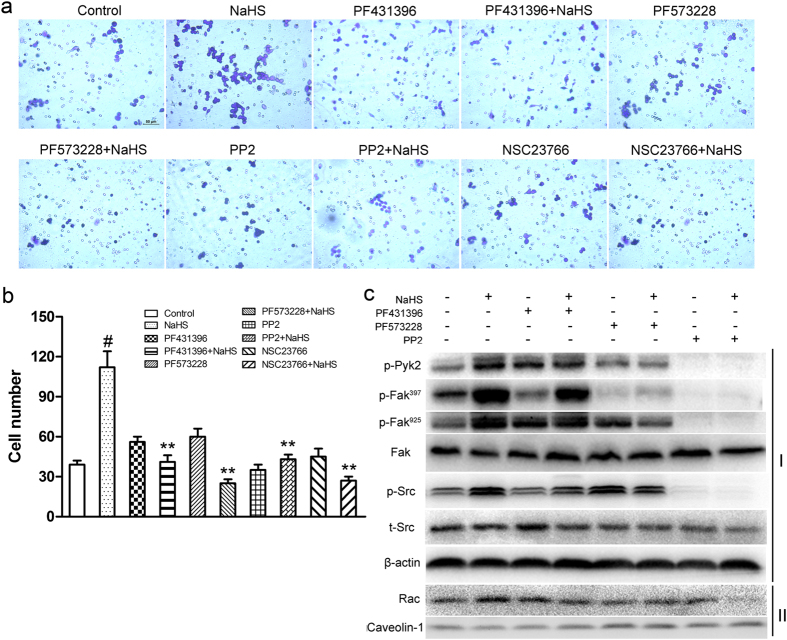
The Src-FAK/Pyk2-Rac pathway responded to macrophage migration triggered by NaHS. (**a**) RAW264.7 cells were incubated for 1 h in the presence or absence of PF431396 (5 μM), PF573228 (5 μM), PP2 (10 μM) or NSC23766 (25 μM) respectively, followed by incubation with additional 100 μM NaHS for 6 h. The migratory ability of RAW264.7 cells was determined by transwell assay. Scale bar, 50 μm. (**b**) The numbers of the migrated cells per field were shown as the mean ± SEM from three independent experiments. ^#^P < 0.05 versus Control, **P < 0.01 versus NaHS treatment. (**c**) RAW264.7 cells were incubated with PF431396 (5 μM), PF573228 (5 μM) or PP2 (10 μM) respectively for 1 h before 100 μM NaHS treatment. The expressions of indicated proteins were assessed by western blot (I, total cell lysates; II, membrane extracts; p, phosphorylated; t, total).

To substantiate the Src-FAK/Pyk2-Rac axis is responsible for H_2_S triggered migration, the RAW264.7 cells were pre-incubated with the Pyk2 inhibitor PF431396, the FAK specific inhibitor PF573228, the Src inhibitor PP2, or the Rac inhibitor NSC23766, and then NaHS was added and incubated for 6 h. The migratory ability of the treated cells was determined by transwell assay. As shown in Fig. 4a,b, NaHS accelerated the migration of RAW264.7 cells, which were diminished by all the four inhibitors. Western blot results were also consistent with the results of transwell assay, revealed that PF431396 suppressed the NaHS-induced activation of Pyk2. Additionally, PF573228 significantly decreased the NaHS triggered phosphorylation of Fak, but had a weak effect on phosphorylated Src. PP2 completely blocked the phosphorylation of Src, Pyk2 and FAK induced by NaHS (Fig. 4c). These results confirm that the Src-FAK/Pyk2-Rac signaling is critical in H_2_S-induced macrophages migration, and also indicate that the direct target of H_2_S is in the up-stream of this signaling pathway.

### NaHS promoted the internalization of macrophage surface integrin β1

**Figure 5:**
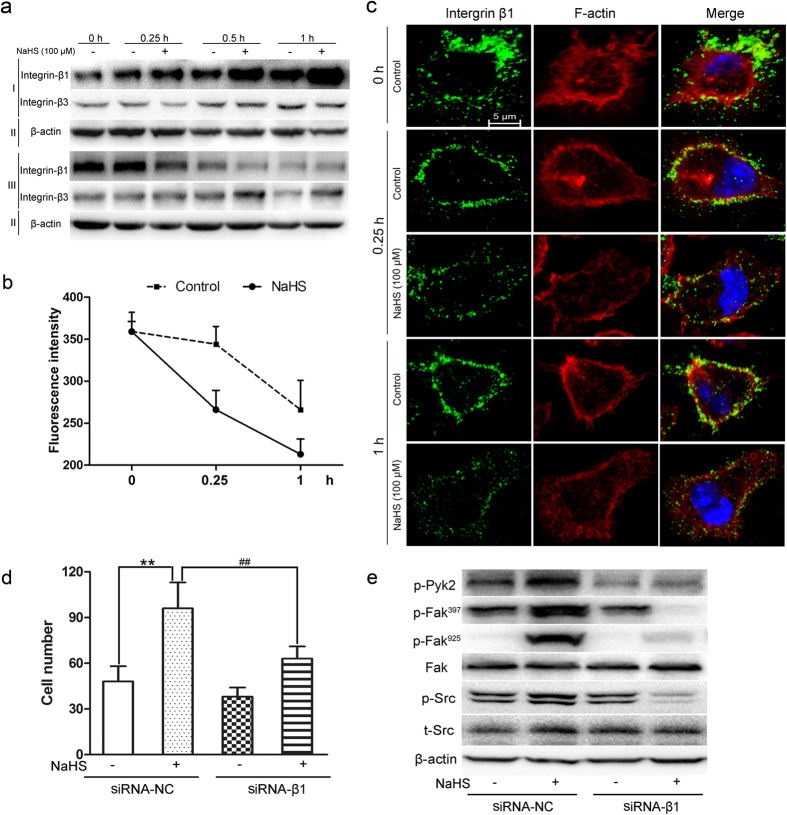
The influence of NaHS in the internalization of surface integrin β1 and Src-FAK/Pyk2 signaling. (**a**) Surface proteins of RAW264.7 cells were biotinylated and separated by streptavidin beads. Integrin β1 and integrin β3 levels were detected by western blot (I: internalized proteins from cell surface; II: total cell lysates; III: cell surface proteins). (**b**) RAW264.7 cells were incubated with NaHS (100 μM) for the indicated time and surface levels of integrin β1 were labeled with the FITC-conjugated secondary antibody, followed by Flow cytometric analysis. (**c**) RAW264.7 cells were incubated with NaHS (100 μM) for the indicated time. Surface Integrin β1 was stained with FITC-conjugated antibody (green); F-actin bundles were detected with Rhodamine (Red); Nuclei were counterstained with DAPI (blue). Scale bar, 5 μm. All the data are representative of three independent experiments. (**d**) After siRNA-integrin β1 or siRNA-NC transfection for 24 h, RAW264.7 cells were incubated without or with NaHS (100 μM) for 6 h, and then the migratory capacities of the cells were evaluated by transwell assay. Values are the mean ± SD from three independent experiments. (**e**) RAW264.7 cells transfected with siRNA-integrin β1 were treated with NaHS (100 μM) for 6 h and the phosphor-Src, -Pyk2, -FAK^397^, -FAK^925^, total Src and FAK were analyzed by western blot (NC, negative control; p, phosphorylated; t, total).

To pinpoint the direct target of H_2_S in triggering macrophage migration, the levels of integrin β1 and β3 in the surface of RAW264.7 cells with or without NaHS treatment were examined. The total level of integrin β1 or β3 did not change within the time course of NaHS treatment (Supplemental Fig. 2). While, the internalization of surface integrin β1 dramatically increased under NaHS treatment. And the surface integrin β1 decreased much more quickly in NaHS treated cells than in control cells. However, only a weak effect was observed on the internalization of integrin β3 (Fig. 5a). In addition, flow cytometric analysis also revealed that the surface integrin β1 was decreased much more quickly following NaHS treatment (Fig. 5b). To further verify the effect of NaHS on the internalization of integrin β1, confocal microscopic analyses were performed. As shown in Fig. 5c, within the indicated time course of treatment, the labeled integrin β1 was gradually internalized in the NaHS groups, but mainly presented on the plasma membrane in the untreated control. Collectively, these findings, on one side, suggest a function of H_2_S in regulating the internalization of macrophage surface integrin β1; on the other side, indicate that H_2_S may activate the Src-FAK/Pyk2-Rac signaling *via* an integrin β1-dependent pathway.

### Integrin β1 silencing inhibited macrophage migration and Src-FAK/Pyk2 signaling activation induced by NaHS

To ascertain that integrin β1 is the target of H_2_S for promoting macrophages migration through activation of Src-FAK/Pyk2-Rac signaling, the integrin β1-specific-siRNA was introduced into RAW264.7 cells. The transfection efficiency was confirmed by western blot, and the data showed that integrin β1 expression in the siRNA-β1 group was reduced significantly (Supplemental Fig. 3). Next, migratory capacity of the siRNA-β1 transfected cells in response to NaHS was evaluated by transwell assay. As shown in Fig. 5d, the increased migratory capacity of H_2_S treated macrophages was significantly impaired by integrin β1-siRNA. Furthermore, the silence of integrin β1 also inhibited the activation of Src-FAK/Pyk2 signaling induced by NaHS (Fig. 5e). Thus, these results confirm that integrin β1-Src-FAK/Pyk2-Rac signaling played a pivotal role in H_2_S-induced macrophage migration.

### Endogenous CSE knockdown diminished macrophage migration, integrin β1 internalization and Src-FAK/Pyk2 signaling activation

**Figure 6:**
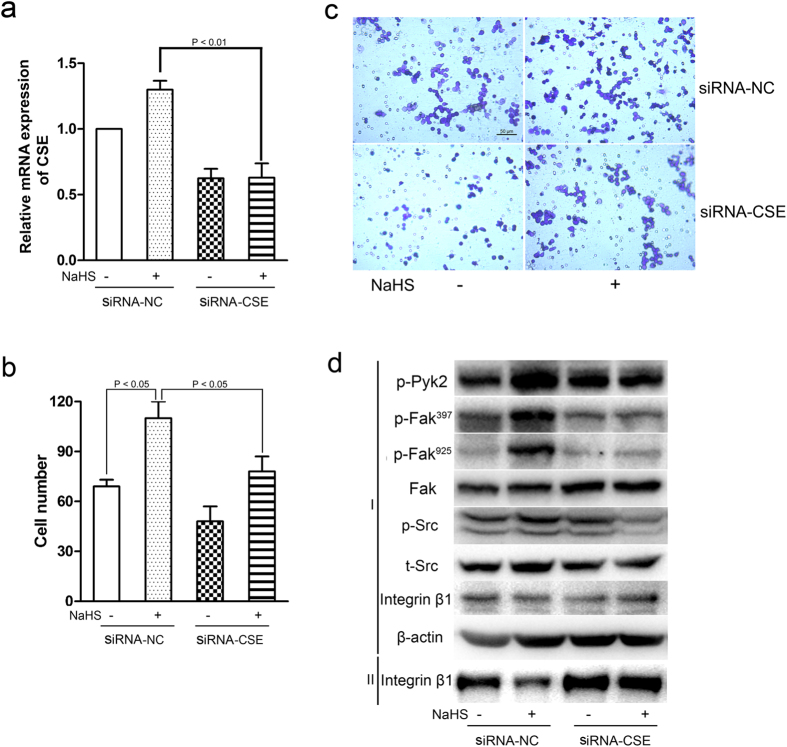
Migration and signaling pathways altered by endogenous CSE silencing in RAW264.7 cells. (**a**) After specific siRNA transfection for 24 h, RAW264.7 cells were treated with NaHS (100 μM) for 6 h, the interference efficiency of CSE was confirmed by quantitative real-time PCR. (**b**,**c**) After siRNA-CSE or siRNA-NC transfection for 24 h, RAW264.7 cells were incubated without or with NaHS (100 μM) for 6 h, and then the migratory capacities of the cells was evaluated by transwell assay. Values are the mean ± SD from three independent experiments. Scale bar, 50 μm. (**d**) RAW264.7 cells transfected with siRNA-CSE were treated with NaHS (100 μM) for 6 h and the expression of phospho-Pyk2, -FAK, total Src and FAK and integrin β1 in total cell lysates (I) or cell surface (II) were analyzed by western blot (NC, negative control; p, phosphorylated; t, total).

To further explore the influence of H_2_S on macrophages migration, endogenous CSE was knocked down by specific siRNA in RAW264.7 cells before the NaHS treatment. Compared to siRNA-NC, the siRNA-CSE group significantly reduced the mRNA level of CSE (Fig. 6a). When the endogenous CSE was down-regulated, the NaHS induced migration of macrophages was abolished (Fig. 6b,c). Additionally, CSE silencing also abrogated the activation of phospho-Src, -Pyk2, and -FAK induced by NaHS. Further, although endogenous CSE knockdown did not alter the total amount of integrin β1, increased the cell surface integrin β1 (Fig. 6d). Taken together, these findings suggest that H_2_S indeed promotes macrophage migration *via* accelerating internalization of integrin β1 and activating the down-stream Src-FAK/Pyk2-Rac signaling.

## Discussion

Up to date, therapeutic effects of clinically applicable drugs for MI are still not satisfactory^1^,^27^. H_2_S as a novel gaseous signaling molecule has been reported a potent cardioprotective effect^16^,^17^. Our previous study showed that increasing endogenous H_2_S by a water-soluble modulator S-Propargyl-Cysteine ameliorated ischemic conditions through angiogenesis promotion^19^. In our recent study, we also proved that decrease of H_2_S by CSE KO aggravated cardiac dysfunction and increased mortality post-MI; while, an improved cardiac remodeling and function accompanied with decreased mortality was observed in both WT mice and CSE KO-MI mice treated with different concentrations of NaHS, suggesting that a significant efficacy of H_2_S for MI treatment (unpublished data).

Macrophages digest debris and dominate inflammation resolution following MI^28^. At the early stage after MI, depletion of infiltrating macrophage aggravates pathological infarct healing, resulting in an increased left ventricular dilatation and wall thinning^9^. Acute MI alters the macrophage phenotype and supply chain^29^. While, increase in macrophage recruitment accelerates infarct repair, and improves cardiac remodeling and function^10^. In the present study, by immunostaining with the antibodies against CD68 and galectin-3, the markers of macrophage^30^,^31^, we found that H_2_S could *in vivo* increase the infiltration of macrophages early after MI, suggesting a protective role of H_2_S in early post-MI inflammation and the subsequent healing process^32^.

Accentuation, prolongation, or expansion of the post infarction inflammatory response leads to a worse remodeling or dysfunction in MI^33^,^34^,^35^. Within the initial stage, MI, on one side, results in the migration of macrophages into the infarcted myocardium, initiating intracellular signaling; on the other side, macrophages responding to signals, could also move from the border zone into the core of an infarct^2^. The phase and rate of macrophage infiltration is orchestrated by a wide range of factors. And yet, the specific signals and mechanism responsible for macrophage migration remain unclear. In our present study, increased infiltrating macrophages raised an important question of how H_2_S triggers the migration of macrophages in the cardiac wound.

Previous report by Frangogiannis showed that macrophage infiltration and migration into the myocardium post-MI involved upregulation of both integrins and adhesion molecules^36^. Integrins, especially β1 and β3, are highly expressed isoforms in macrophages, and have been reported to play important roles in receiving ECM signals and regulating cell migration^37^,^38^,^39^. Integrin trafficking, including internalization, is the crucial step in cell communication^38^. Downregulation of integrin β1 at the plasma membrane is a potential mechanism for enhancing cell motility^40^. Thus, through controlling integrin β1 internalization and recycling, junctional adhesion molecule could promote neutrophil chemotaxis^41^. Here, by biotin-based experiments, we found that H_2_S dramatically induced the internalization of integrin β1, but not integrin β3; and silence of integrin β1 inhibited the migration of macrophages, indicating that integrin β1 might be the possible target of H_2_S in regulating macrophage migration.

The internalization of integrin β1 can activate down-stream FAK/Src signaling, which is an important pathway involving in cell migration. The expression or activation of Src, a cellular nonreceptor tyrosine kinase in the downstream of integrin signaling, could reflect the migratory ability of macrophage^42^. As a substrate of Src, FAK serves as an adaptor that connects integrins with actin cytoskeleton^43^. Upon integrin activation, FAK is phosphorylated at tyrosine residues Tyr^397^ or/and Tyr^925^, which in turn activates the binding site Src^42^,^43^,^44^. Recently, Liu at al proved that integrins, phospho-FAK and phospho-Src participate in MI microenvironment improvement for myocardial repair^45^. By analogy to the related FAK kinase, Pyk2 is important in macrophage activation^46^. Additionally, evidence also shows that through controlling phosphorylation of Pyk2 and paxillin, Pyk2 regulates the polarization, migration, and spreading of macrophage^47^. Rac, a member of Rho GTPase family, its activation is also related to the cell polarization and migration in macrophages^48^. In this study, the phospho-Src, -Pyk2, -FAK^397^, and -FAK^925^ were significantly upregulated upon H_2_S treatment, and the downstream Rac was activated relatively later. Inhibitors for FAK, Src, Pyk2, and Rac could effectively diminish the migratory ability of macrophages stimulated by H_2_S and block the activation of related signaling molecules in response to H_2_S. Those results hint that H_2_S triggers macrophage migration *via* the Src-FAK/Pyk2-Rac axis.

In conclusion, our results revealed that H_2_S treatment exerted an increased infiltration of macrophages into the infarcted myocardium at early stage of MI in both WT mice and CSE-KO mice, and the increased macrophage infiltrating antagonized the damage of cardiomyocytes induced by hypoxia. The favorable effects of H_2_S on the infarcted myocardium can be attributed to the promotion of infiltrating macrophages. Further, we present evidence for a novel mechanism that macrophage migration triggered by H_2_S is mediated by integrin β1-Src-FAK/Pyk2-Rac pathway. Thus, activation of macrophages by H_2_S supplement can be a promising therapeutic strategy for MI treatment.

## Additional Information

**How to cite this article**: Miao, L. *et al*. Hydrogen Sulfide Recruits Macrophage Migration by Integrin β1-Src-FAK/Pyk2-Rac Pathway in Myocardial Infarction. *Sci. Rep*. **6**, 22363; doi: 10.1038/srep22363 (2016).

## Supplementary Material

Supplementary Information
