# Author Correction: Hydrogen Sulfide Recruits Macrophage Migration by Integrin β1-Src-FAK/Pyk2-Rac Pathway in Myocardial Infarction

**DOI:** 10.1038/s41598-022-06168-w

**Published:** 2022-02-02

**Authors:** Lei Miao, Xiaoming Xin, Hong Xin, Xiaoyan Shen, Yi-Zhun Zhu

**Affiliations:** 1 Department of Pharmacology, School of Pharmacy and Institutes of Biomedical Sciences, Fudan University, Shanghai, China; 2 Department of Pharmacology, School of Pharmacy, Macau University of Science & Technology, Macau, China

Correction to: *Scientific Reports* 10.1038/srep22363, published online 02 March 2016

This Article contains errors in Figures 1 and 4.

In Figure 1, the Day 8 images for the MI tissues from the KO Mice are a duplication of the Day 3 images for the MI tissues from the KO Mice. Additionally, in Figure 4, panel A, the image for NSC23766 + NaHS is a duplication of PF573228 + NaHS.

The correct Figures 1 and 4 and accompanying legends appear below.

**Figure 1:**
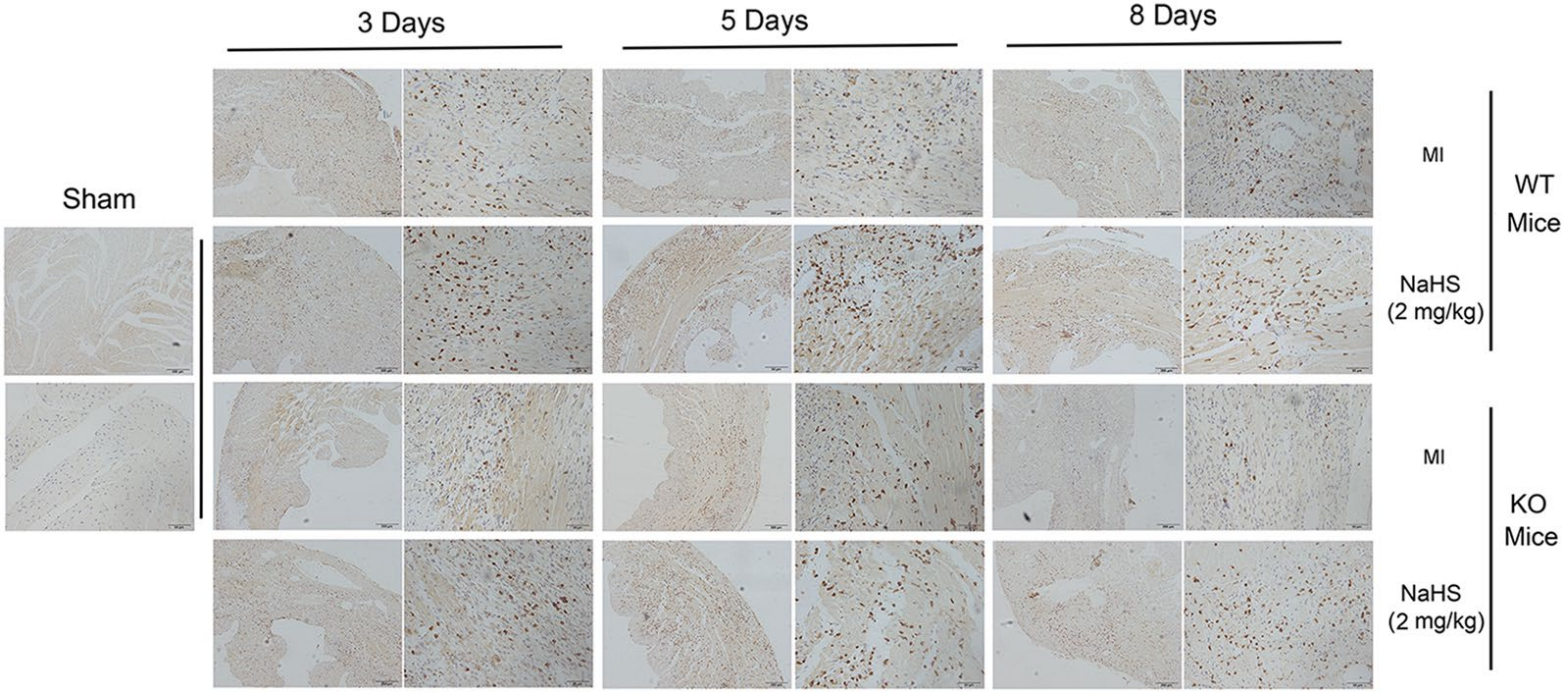
Myocardial immunostaining of CD68 after 3, 5, or 8 days of post-MI treatment with NaHS in both WT and KO mice. Scale bars, 200 μm and 500 μm.

**Figure 4:**
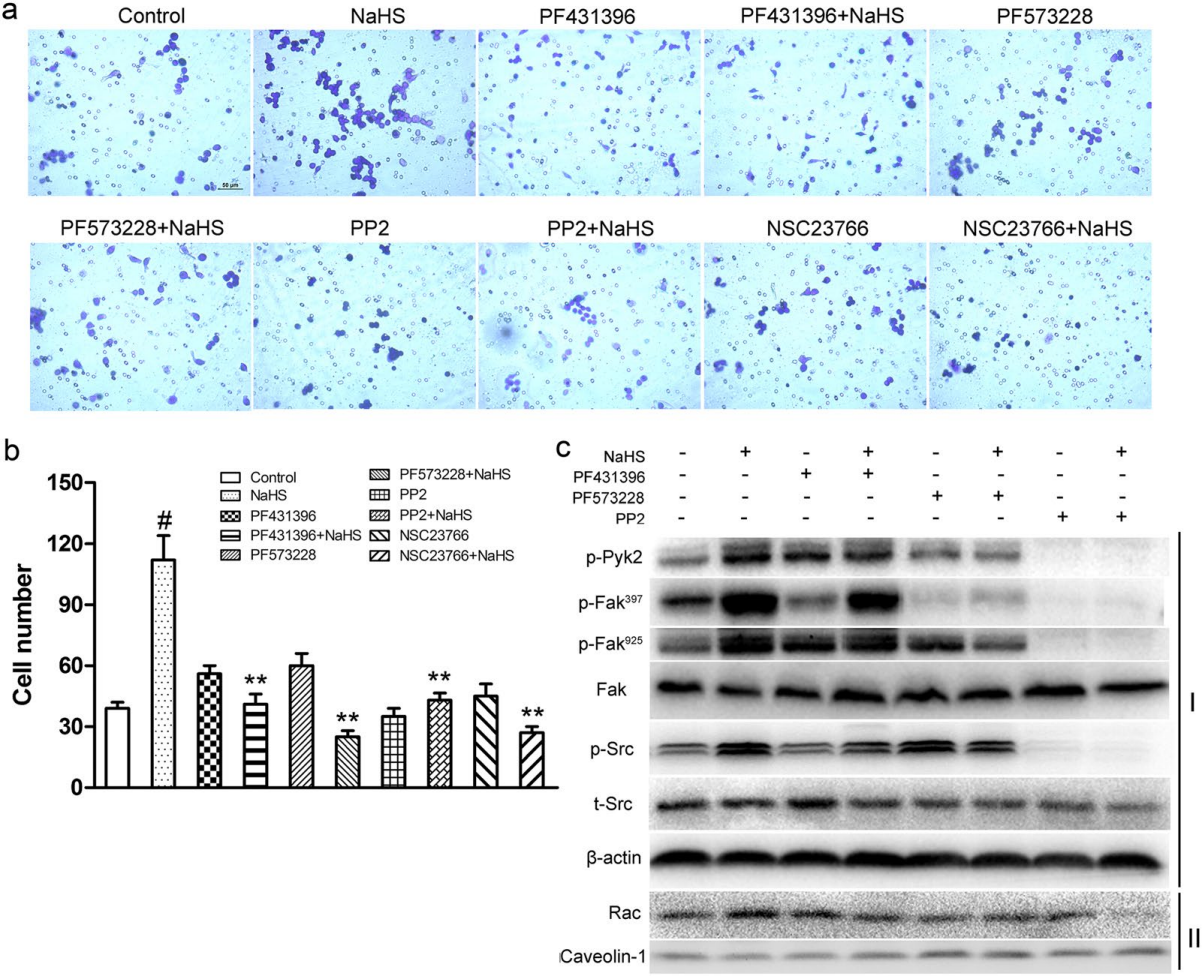
The Src-FAK/Pyk2-Rac pathway responded to macrophage migration triggered by NaHS. (**a**) RAW264.7 cells were incubated for 1 h in the presence or absence of PF431396 (5 μM), PF573228 (5 μM), PP2 (10 μM) or NSC23766 (25 μM) respectively, followed by incubation with additional 100 μM NaHS for 6 h. The migratory ability of RAW264.7 cells was determined by transwell assay. Scale bar, 50 μm. (**b**) The numbers of the migrated cells per field were shown as the mean ± SEM from three independent experiments. ^#^*P* < 0.05 versus Control, **P < 0.01 versus NaHS treatment. (**c**) RAW264.7 cells were incubated with PF431396 (5 μM), PF573228 (5 μM) or PP2 (10 μM) respectively for 1 h before 100 μM NaHS treatment. The expressions of indicated proteins were assessed by western blot (I, total cell lysates; II, membrane extracts; p, phosphorylated; t, total).

