# Supplementary material for: Hydrogen Sulfide Recruits Macrophage Migration by Integrin β1-Src-FAK/Pyk2-Rac Pathway in Myocardial Infarction
Source: Sci Rep. 2016 Mar 2;6:22363. doi: 10.1038/srep22363 (PMC4773762; doi:10.1038/srep22363)
Supplement: Supplementary Information [file srep22363-s1.pdf]

Hydrogen Sulfide Recruits Macrophage Migration by Integrin β1-Src-FAK/Pyk2-Rac Pathway in Myocardial Infarction

Lei Miao[1,]*, Xiaoming Xin[1,]*, Hong Xin[1], Xiaoyan Shen[1] & Yi-Zhun Zhu[1, 2]

[1]Department of Pharmacology, School of Pharmacy and Institutes of Biomedical Sciences, Fudan University, Shanghai, China. [2]Department of Pharmacology, Loo Yong Lin School of Medicine, National University of Singapore, Singapore, Singapore. *These authors contributed equally to this work. Correspondence and requests for materials should be addressed to Y.Z. Z. (zhuyz@fudan.edu.cn), or X.Y. S. (shxiaoy@fudan.edu.cn)

Supplementary Figures

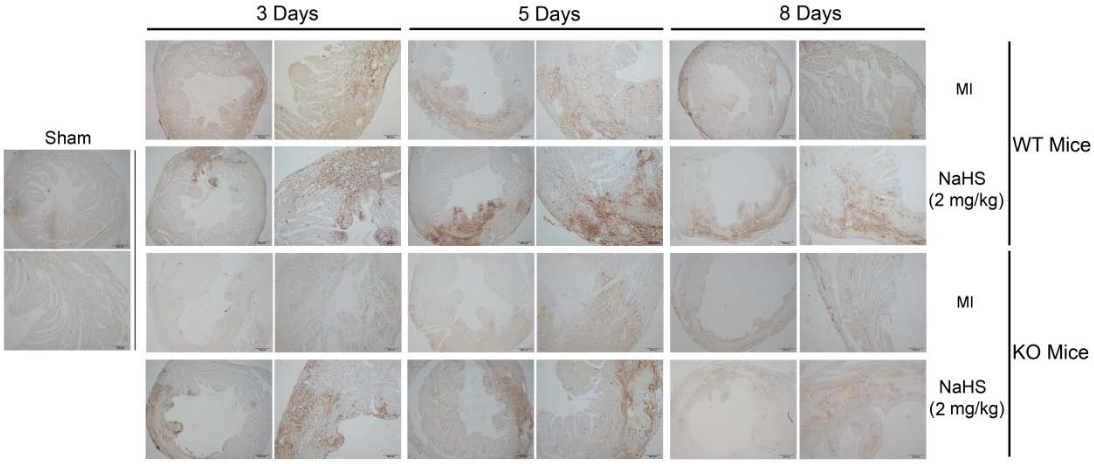

Figure 1. Myocardial immunostaining of galectin-3 after 3, 5, or 8 days of post-MI treatment with NaHS in both WT and KO mice. Scale bars, 200 μm and 500 μm.

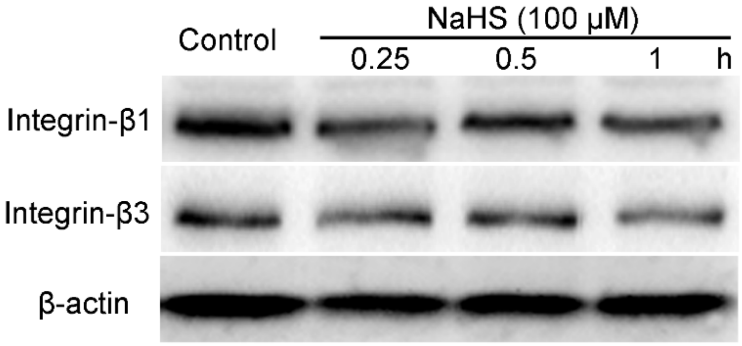

Figure 2. Total proteins were extracted from RAW264.7 cells treated with NaHS for indicated time, and the expression of Integrin β1 and integrin β3 were analyzed by western blot.

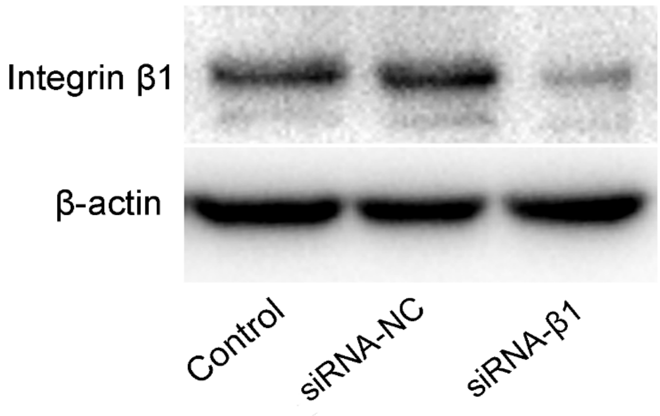

Figure 3. The expression of integrin β1 after specific siRNA transfection for 24 h in RAW264.7 cells was determined by western blot.
